# Development and Host Compatibility of Plasmids for Two Important Ruminant Pathogens, *Mycoplasma bovis* and *Mycoplasma agalactiae*

**DOI:** 10.1371/journal.pone.0119000

**Published:** 2015-03-06

**Authors:** Shukriti Sharma, Chistine Citti, Eveline Sagné, Marc S. Marenda, Philip F. Markham, Glenn F. Browning

**Affiliations:** 1 Asia-Pacific Centre for Animal Health, Faculty of Veterinary and Agricultural Sciences, The University of Melbourne, Parkville, Victoria, 3010, Australia; 2 INRA, ENVT, UMR 1225, 31076, Toulouse, France; Louisiana State University and A & M College, UNITED STATES

## Abstract

*Mycoplasma bovis* is a cause of pneumonia, mastitis, arthritis and otitis media in cattle throughout the world. However, despite its clinical significance, there is a paucity of tools to genetically manipulate it, impeding our capacity to further explore the molecular basis of its virulence. To address this limitation, we developed a series of homologous and heterologous replicable plasmids from *M*. *bovis* and *M*. *agalactiae*. The shortest replicable *oriC* plasmid based on the region downstream of *dnaA* in *M*. *bovis* was 247 bp and contained two DnaA boxes, while *oriC* plasmids based on the region downstream of *dnaA* in *M*. *agalactiae* strains 5632 and PG2 were 219 bp and 217 bp in length, respectively, and contained only a single DnaA box. The efficiency of transformation in *M*. *bovis* and *M*. *agalactiae* was inversely correlated with the size of the *oriC* region in the construct, and, in general, homologous *oriC* plasmids had a higher transformation efficiency than heterologous *oriC* plasmids. The larger pWholeoriC45 and pMM21-7 plasmids integrated into the genomic *oriC* region of *M*. *bovis*, while the smaller *oriC* plasmids remained extrachromosomal for up to 20 serial passages in selective media. Although specific gene disruptions were not be achieved in *M*. *bovis* in this study, the *oriC* plasmids developed here could still be useful as tools in complementation studies and for expression of exogenous genes in both *M*. *bovis* and *M*. *agalactiae*.

## Introduction

*Mycoplasma bovis* is a major cause of chronic caseonecrotic bronchopneumonia in calves [1,2]. Its contribution to bovine respiratory tract disease is commonly complicated by concurrent infection with a number of viral and bacterial pathogens, including bovine herpesvirus type 1, parainfluenza virus type 3, bovine respiratory syncytial virus, bovine pestivirus, bovine adenovirus, *Mannheimia haemolytica*, *Pasteurella multocida* and *Histophilus somni*. *M*. *bovis* is also an important cause of mastitis and lowered milk production, and can cause arthritis, otitis media, keratoconjunctivitis, meningitis, abortion and infertility [3,4]. It has been reported to be an emerging global pathogen [3–6], with estimated annual losses of €144–192 million attributed to its contribution to bovine pneumonia in Europe [4] and of $108 million to its contribution to bovine mastitis in the USA [7].

The genome sequence of the *M*. *bovis* type strain PG45, as well as the Hubei-1 and HB0801 strains, have been completed and published recently [8–10], but the paucity of genetic tools for generating targeted gene knockouts and for complementation studies impedes our ability to examine gene function in *M*. *bovis*. Transposon mutagenesis using Tn*916* and Tn*4001* and their derivatives has been used to study the genetics of several human and animal mycoplasmas, but the random insertion of a transposon in the genome of an organism does not allow specific targeting of a gene of interest [11,12].

Although homologous recombination with linear DNA has been achieved occasionally in some mycoplasmas, targeted gene disruption through homologous recombination remains the main alternative to transposons for mutagenesis studies. The classical method of using non-replicable plasmids to disrupt gene targets through homologous recombination has been successful only in a few mollicutes, including *Acholeplasma laidlawii* [13], *M*. *gallisepticum* [14–16] and *M*. *genitalium* [17,18], probably because the likelihood of allelic exchange is reduced by the low transformation efficiencies and low recombination frequencies seen in mycoplasmas.

Alternatively, replicable plasmids containing compatible origins of replication (*oriC*) have been developed [19]. Replicable *oriC* plasmids have multiple uses, including inactivation of target genes, expression of foreign genes and complementation of mutated organisms [15,20–24]. They have been developed for a number of mollicutes [25], including *Spiroplasma citri*, *M*. *mycoides* subspecies *mycoides*, *M*. *mycoides* subspecies *capri*, *M*. *capricolum* subspecies *capricolum*, *M*. *pulmonis*, *M*. *gallisepticum*, *M*. *hyopneumoniae* and *M*. *agalactiae*, but they are not available for *M*. *bovis*.

In the present study, a series of plasmids containing the *oriC* regions of *M*. *bovis* and *M*. *agalactiae* fused with a tetracycline resistance marker were developed. Using these constructs functional analyses of homologous and heterologous *oriC* plasmids were conducted in *M*. *bovis* strain PG45 and *M*. *agalactiae* strain PG2 to determine the optimal *oriC* regions to include in plasmid constructs for these two species.

## Materials and Methods

### Bacterial strains and culture conditions

The *M*. *bovis* type strain PG45 was grown at 37°C in modified Frey’s broth or on mycoplasma agar plates [26], while the *M*. *agalactiae* type strain PG2 was grown at 37°C in Aluotto broth or on Aluotto agar [27]. *M*. *bovis* and *M*. *agalactiae* transformants were selected on plates containing tetracycline at 5 μg/ml and 2 μg/ml (Sigma Aldrich), respectively. *E*. *coli* DH5α cells (Life Technologies) were used for subcloning and for amplifying different *oriC* plasmids.

### Construction of *oriC* plasmids from *M*. *bovis*

Several regions of the predicted *oriC* region of *M*. *bovis* strain PG45 were amplified by PCR to generate the plasmids pWholeoriC45, pIRR45 and pIRL45 using appropriate oligonucleotide primers (Invitrogen), as listed in Table 1. Initially an *oriC* region including the *dnaA* gene and both intergenic regions, designated whole *oriC* (1.8 kbp), was amplified using the primers OMBIRL for/OMBIRR rev. The left (IRL) and right intergenic (IRR) regions were amplified separately, without the intervening *dnaA* gene, using the primer pairs OMBIRL for/OMBIRL rev and OMBIRR for/OMBIRR rev, respectively. Each 50 μl PCR contained 1 x Hi Fi reaction buffer, 200 μM of each deoxynucleotide triphosphate, 2 mM MgSO_4_, 250 nM of each primer, 2.5 U Platinum Taq High Fidelity DNA polymerase (Invitrogen), template DNA, and water to make the final volume up to 50 μl.

**Table 1:** Oligonucleotide pairs used to generate and assess *oriC* plasmids.

Oligonucleotides	Sequence (5’-3’)	PCR product (size)	Reference (nucleotide sequence)
OMBIRL for	tcttgattactgttgcttga	IRL region of *M*. *bovis* (237 bp)	NC_014760.1 (1003214 to 0000048)
OMBIRL rev	aaagcaatttccttatcatt		
OMBIRR for	caacgagatttttaagaaaag	IRR region of *M*. *bovis* and oriC probe (247 bp)	NC_014760.1 (1385 to 1631)
OMBIRR rev	ccatataaagaactaattgga		
OMBIRL for	tcttgattactgttgcttga	Whole *oriC* of *M*. *bovis* (1.8 kbp)	NC_014760.1 (1003214 to 0001631)
OMBIRR rev	ccatataaagaactaattgga		
OMAIRR for	gataagcaacgagatttttaag	IRR regions of *M*.*agalactiae* (219 bp in MAG5632; 217 bp in MAGPG2)	CU179680.1 (1371 to 1587); FP671138.1 (1371–1589)
OMAIRR rev	ttgaaacaacttcgataatgtca		
IRROMA133 for	cattttaaaaagcggttttaaac	IRR133 region of MAG5632 (133 bp)	FP671138.1 (1402 to 1534)
IRR MA66 rev	attttccttaattaataaatatatg		
IRROMA90 for	GGGCCCaaaaattgtattttttgttac	IRR90 region of MAG5632 (102 bp)	FP671138.1 (1463 to 1570)
IRROMA90 rev	GACGTCtaaaaaatttttgtttat		
IRROMA 66for	aaaaattgtattttttgttacttatc	IRR66 region of MAG5632 (66 bp)	FP671138.1 (1469 to 1534)
IRROMA 66 rev	attttccttaattaataaatatatg		
IRR OMA38 for	ttatccacaaattaacaaaacatatatttattaattaa	IRR38 region of MAG5632 (38 bp)	FP671138.1 (1490 to 1527)
IRR OMA38 rev	ttaattaataaatatatgttttgttaatttgtggataa		
LAtetM for	gcagttatggaagggatacg	TetR screening PCR and probe (339 bp)	Sharma *et al*. (2014)
LBtetM rev	ttcttgaatacaccgagcag		
AmpR for	ccaatgcttaatcagtgagg	AmpR probe (862 bp)	Lee *et al*. (2008)
AmpR rev	gtatgagtattcaacatttccg		
GKp48 for	GCGGCCGCttgctgcttcatgtggtgat	Internal *p48* fragment (392 bp)	NC_014760.1 (13995 to 14386)
GKp48 rev	CTGCAGgcgctgctttgtgagtaaatc		
GKRE for	GCGGCCGCtgttgaaacattattaccaacaaaca	Internal *typeII RE* fragment (462 bp)	NC_014760.1 (648209 to 648670)
GKRE rev	CTGCAGtcgcccatgtgtatctaaacc		
GKXer1 for	GCGGCCGCttgcagcatataaaaacatacttgc	Internal *xer1* fragment (251 bp)	NC_014760.1 (948459 to 948709)
GKXer1 rev	taCTGCAGtgtgcctttgtgagaataggtc		
GKoriCrecA for	CTGCAGaagttcgaaaaaatccaattacacaaaatt	*recA* gene (1.6 kbp)	CP001873.1 (447765 to 449275)
GKoriCrecA rev	agGTCGACttattggcaatcttttaacttatttaatacc		

Upper case letters indicate the restriction endonuclease cleavage sites incorporated into the oligonucleotide primer

TetR = tetracycline resistance gene, AmpR = ampicillin resistance gene, *typeII RE* = type II restriction endonuclease gene

For amplification of the whole *oriC* region, 20 ng of genomic DNA was used as template and the reaction was incubated in a thermocycler (iCycler, Bio-Rad) at 94°C for 5 min, then through 40 cycles of 94°C for 30 sec, 50°C for 2 min and 68°C for 4 min, and finally at 68°C for 7 min. For amplification of the IRL and IRR regions, the DNA template was prepared by pelleting 1 ml of culture of *M*. *bovis* PG45 and resuspending the cells in 100 μl distilled water, then heating the suspension at 95°C for 5 min. A 2 μl volume of this was used as template in the PCR reaction. For amplification of the intergenic regions, the conditions were: 95°C for 2 min, followed by 35 cycles of 95°C for 30 sec, 50°C for 30 sec and 68°C for 20 sec, with a final extension at 68°C for 5 min.

**Fig 1:**
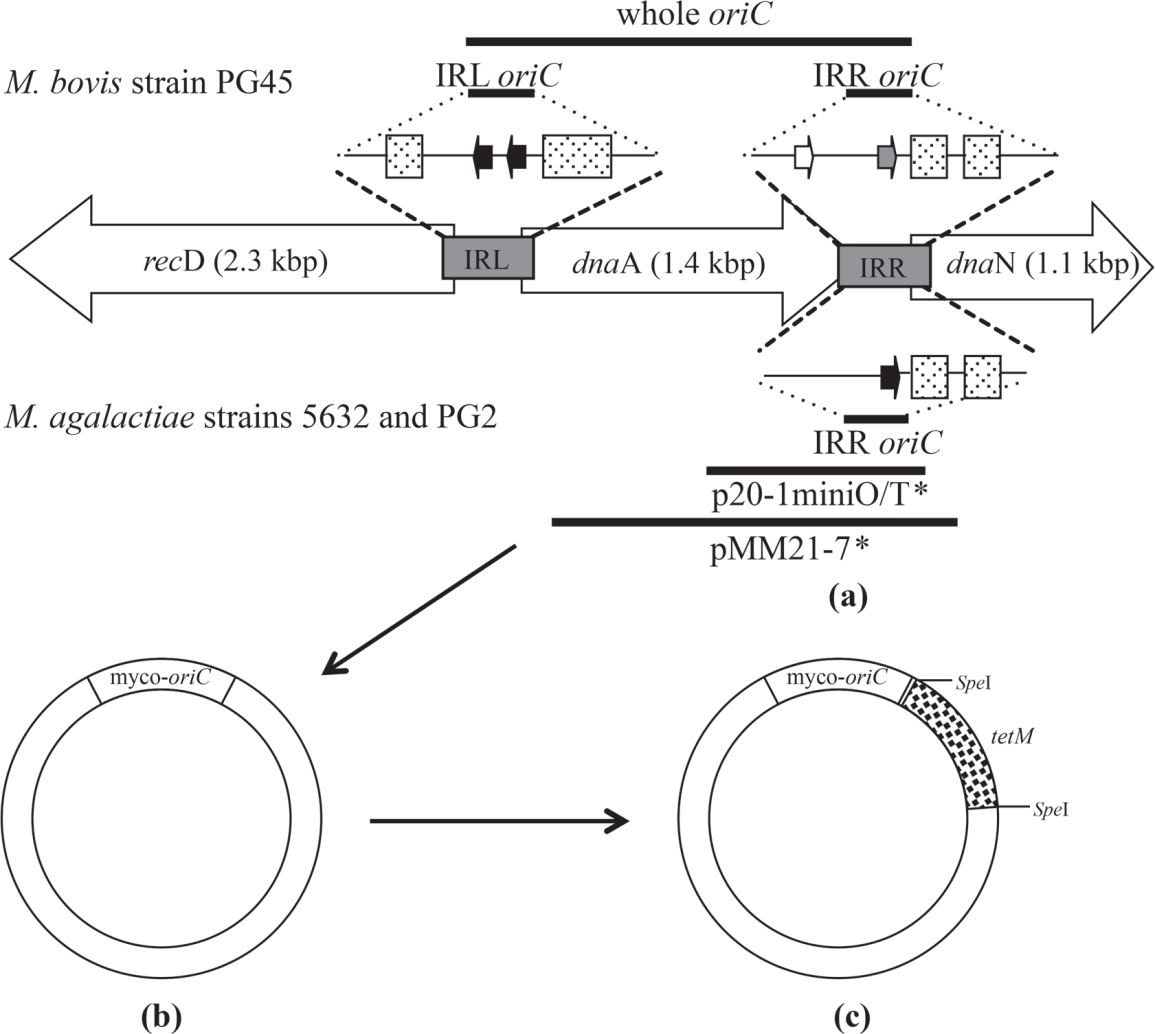
Construction of homologous and heterologous *oriC* plasmids in a pGEM-T backbone. (a) Solid lines indicate the amplified *oriC* regions, shaded rectangles indicate the location of AT rich clusters, while arrows indicate the location and nucleotide similarity of DnaA boxes to the consensus sequence: black 9, grey 8 and white 7, of 9 nucleotides identical. The whole *oriC* region containing *dnaA* with the left and right intergenic regions, as well as left (IRL) and right intergenic regions (IRR) wasamplified from *M*. *bovis* PG45 strain, while the IRR regions were also amplified from the *M*. *agalactiae* strains PG2 and 5632. The *oriC* plasmids marked with an asterisk (*) were developed in a pBluescript KS vector backbone, and had been used previously for transformation of *M*. *agalactiae*. (b) Amplified *oriC* products with varying *oriC* regions were ligated into the multicloning site of pGEM-T (Promega). (c) The tetracycline resistance gene (*tetM*) was then cloned into the *Spe*I site of these plasmids.

PCR products were separated by agarose gel electrophoresis, the expected bands excised and DNA extracted from the gel slices. All the products were ligated separately into the T/A-cloning site (Fig. 1) of pGEM-T (Promega), the clones selected on the basis of blue-white screening and the sequence of the insert confirmed by DNA sequencing using ABI PRISM Big Dye 3.1 Terminator chemistry (Applied Biosystems). The tetracycline resistance gene (*tetM*) with its own promoter and terminator was digested from the plasmid pMlori [15] using the restriction endonuclease *Spe*I (New England Biolabs) and ligated into the *Spe*I cleavage site of the pGEM-T construct.

### Construction of *oriC* plasmids from *M*. *agalactiae*

The heterologous *oriC* plasmid pMM21-7 containing the 1.3 kbp *oriC* region of *M*. *agalactiae* strain 5632 [28], developed for transformation of *M*. *agalactiae*, was initially used to transform *M*. *bovis*. Subsequently, plasmid p20-1miniO/T [20] with a reduced *oriC* region (700 bp) from *M*. *agalactiae* strain 5632 was used to transform *M*. *bovis* and *M*. *agalactiae*.

To determine the minimal replicable *oriC* region of *M*. *agalactiae*, pIRR5632 and pIRRPG2 constructs were developed based on the IRR regions of *M*. *agalactiae* strains 5632 and PG2, respectively, using the primers OMAIRR for/OMAIRR rev (Table 1). The PCR composition and thermocycling conditions were similar to those used for amplifying the intergenic regions of *M*. *bovis*. The amplified IRR regions were cloned separately into the T/A-cloning site (Fig. 1) of pGEM-T and the *tetM* gene was then ligated into the *Spe*I cleavage site of the vector. To examine whether the size of the *oriC* region capable of replication could be reduced further, several *oriC* regions were amplified (S1 Fig.) using plasmid pIRR5632 as template. These included the 133 bp intergenic region, as well as regions with only a single DnaA box and nearby AT rich clusters. The amplification of these 133, 90 and 66 bp regions was performed with the primers listed in Table 1 and reactions were incubated at 95°C for 3 min, followed by 35 cycles of 95°C for 30 sec, 45°C for 20 sec and 68°C for 20 sec, with a final extension at 68°C for 5 min, while a 38 bp region was generated by oligonucleotide annealing, starting at 95°C for 3 min then slowly cooling to 25°C over a 45 min period. These regions were cloned separately into pGEM-T and the *tetM* gene was then ligated into the construct as described above.

### Transformation of *M*. *bovis* and *M*. *agalactiae*

Approximately 5 μg of DNA of each plasmid was introduced into *M*. *bovis* strain PG45 cells at late log phase by electroporation [26,29], with 5 μg tetracycline/ml used for selection in broth cultures and on agar plates.

Competent *M*. *agalactiae* strain PG2 cells were prepared from late log phase cultures. For transformation, 3 μg of DNA of each plasmid was mixed with 20 μl electrocompetent cells (approximately 10^8^ cells) and 20 μl ice-cold HEPES-sucrose buffer (8 mM HEPES, 272 mM sucrose, pH 7.4) in a Gene Pulser cuvette (Bio-Rad) with a 0.2 cm electrode gap. The remainder of the transformation protocol was similar to that used for *M*. *bovis* strain PG45, with the exception that tetracycline was used at a concentration of 2 μg/ml in Aluotto broth and plates.

### Determination of transformation efficiency

To calculate the efficiency of transformation with different *oriC* plasmids, a sample of the transformed culture was taken after 2 h incubation at 37°C in non-selective broth and inoculated onto an agar plate to assess the total number of colony forming units (CFU) in the absence of antibiotic selection, and the remaining cells were plated onto selective agar plates containing an appropriate amount of tetracycline. To confirm the presence of the plasmid, a *tetM* screening PCR [26] was performed with the primers LAtetM for and LBtetM rev (Table 1). In the presence of free or integrated *oriC* plasmids, this PCR generated a 339 bp *tetM* product that could be detected following agarose gel electrophoresis.

### Design of constructs to engineer gene knockouts

To construct an *oriC* plasmid that could integrate into a target gene by homologous recombination, internal fragments of *xer1*, and the p48 and the type II restriction endonuclease genes were amplified separately from *M*. *bovis* (Table 1), with *Not*I and *Pst*I restriction endonuclease cleavage sites incorporated into the forward and reverse primers, respectively, and inserted into the IRR-based *oriC* plasmids (S2 Fig.). For amplification of 392 and 462 bp internal fragments of the p48 and type II restriction endonuclease genes, the PCR was incubated through one cycle of 94°C for 3 min, followed by 35 cycles of 94°C for 45 sec, 55°C for 45 sec and 68°C for 30 sec, with a final extension at 68°C of 5 min, while the amplification of the 251 bp *xer1* internal fragment was carried out by incubation through one cycle of 94°C for 2 min, followed by 35 cycles of 94°C for 30 sec, 50°C for 30 sec and 68°C for 20 sec, with a final extension of 68°C for 5 min. A heterologous *recA* gene, along with its promoter and terminator, was amplified from *M*. *gallisepticum* strain S6 with the primer pair GKoriCrecA for/GKoriCrecA rev (Table 1) and ligated between the *Pst*I and *Sal*I cleavage sites (S2 Fig.) in *oriC* plasmids that already contained engineered gene fragments. The amplification reaction was incubated through one cycle of 94°C for 5 min, followed by 40 cycles of 94°C for 30 sec, 50°C for 30 sec and 68°C for 1.4 min, with a final extension at 68°C for 7 min. The *xer1* and p48 gene fragments were inserted into pIRR45, while the type II restriction endonuclease gene fragment was introduced into pIRRPG2.

### DNA isolation, digestion and Southern hybridisation

To evaluate chromosomal integration, the presence of the free replicating form of the plasmids and gene target disruption in *M*. *bovis*, independent transformants were selected and passaged in selective media containing tetracycline at 5 μg/ml. DNA from *M*. *bovis* strain PG45 was isolated by phenol-chloroform extraction [30]. Control plasmid DNA was isolated from *E*. *coli* DH5α cells and total genomic DNA from transformant cells was extracted using the High Pure PCR template preparation kit (Roche).

Approximately 3 μg of genomic DNA from the wild type and *M*. *bovis* strain PG45 transformants was digested at 37°C for 16 h. After separation in a 0.8% agarose gel containing 0.1 μg ethidium bromide/ml, the fragments were transferred to Hybond-N^+^ nylon membrane (GE Healthcare) by capillary action [30]. The membrane was washed with 6 x SSC (1 x SSC consists of 150 mM NaCl, 15 mM sodium citrate, pH 7.0) for 10 min, air-dried and the transferred DNA fixed to the membrane by exposure to UV light for 4 min. The membrane was prehybridised with 15 ml of DIG Easy Hyb hybridisation buffer (Roche) in a roller bottle at 42°C for 30 min in a hybridisation oven (Hybaid). Approximately 250 ng of the digoxigenin (DIG)-labelled probe was denatured at 95°C for 5 min, immediately cooled on ice, then added to the hybridisation buffer and incubated with the membrane overnight. The membrane was washed twice in low stringency wash buffer (2 x SSC, 0.1% SDS) at RT for 5 min each and then twice in higher stringency buffer (0.5 x SSC, 0.1% SDS) at 65°C for 15 min each. After cooling to RT, the membrane was washed briefly in washing buffer (0.1 M maleic acid, 0.15 M NaCl, pH 7.5, and 0.3% v/v Tween-20), incubated for 30 min in 1 x blocking buffer (1% w/v blocking reagent in maleic acid buffer) at RT, then a dilution of 1:5000 of an anti-DIG antibody coupled to alkaline phosphatase (Roche) in 1 x blocking buffer added and incubated with the blot for 30 min at RT. The membrane was washed twice in washing buffer for 30 min each at RT and then equilibrated in detection buffer (0.1 M Tris-HCl, 0.1 M NaCl, pH 9.5) for about 5 min. Hybridisation was detected by addition of the chemiluminescent substrate disodium 3-(4-methoxyspiro {1,2-dioxetane-3,2’-(5'-chloro)tricyclo[3.3.1.13,7]decan}-4-yl) phenyl phosphate (CSPD, Roche) as recommended by the manufacturer and exposing the membrane to X-ray film (Kodak), which was developed using an Agfa automated film processor.

Southern blot hybridisation analysis was performed with DIG-labelled probes targeting the 247 bp IRR *oriC* region of *M*. *bovis* strain PG45 (*oriC* probe), the 339 bp *tetM* region or the 862 bp ampicillin resistance gene (*ampR*) region, and overnight prehybridisation was performed either at 40°C (*oriC* probe) or 46°C (*tetM* and *ampR* probe) and a high stringency wash step of 15 min each was performed twice at 62°C (*oriC* probe) or 65°C (*tetM* and *ampR* probes).

## Results

### Replicability of homologous and heterologous *oriC* plasmids

*M*. *bovis* strain PG45 was transformed with the homologous *oriC* plasmid pWholeoriC45, containing the *dnaA* gene and its upstream and downstream intergenic regions along with the *tetM* determinant, which confers tetracycline resistance in Mollicutes. As a control, *M*. *agalactiae* strain PG2 was transformed with the homologous *oriC* plasmid pMM21-7 [28], containing the 1.3 kbp *oriC* region of *M*. *agalactiae* strain 5632. To examine the replicability of heterologous *oriC* plasmids in *M*. *bovis and M*. *agalactiae*, the pMM21-7 plasmid or its derivative p20-1miniO/T were used to transform *M*. *bovis* strain PG45 cells, while *M*. *agalactiae* strain PG2 cells were transformed with the pWholeoriC45 plasmid.

Mycoplasma cells transformed with the homologous and heterologous constructs generated colonies on selective agar plates containing an appropriate concentration of tetracycline. The individual clones were selected and grown in appropriate selective broth. PCR analyses confirmed that tetracycline resistance was correlated with the presence of the *tetM* determinant in all the transformants, as detectable by amplification of a predicted 339 bp PCR product. These results indicated that the *oriC* region capable of replicating in *M*. *bovis* is located within the *dnaA* gene or its flanking regions.

### Minimal functional *oriC* region

In order to determine the smallest *oriC* region of *M*. *bovis* required for plasmid replication, *M*. *bovis* and *M*. *agalactiae* cells were transformed with the pIRL45 and pIRR45 plasmids, both of which lacked the *dnaA* gene (Fig. 1). The pIRR45 plasmid, which contained the IRR region downstream of *dnaA*, was consistently able to transform both species, whilst the pIRL45 plasmid, which contained the region upstream of the *dnaA* gene, was repeatedly unable to transform *M*. *bovis* (Table 2). The *oriC* region of pIRR45 consisted of a 247 bp region with two AT rich regions of 17 bp each and two putative DnaA boxes that differ from the consensus sequence [TTATCCACA] by one or two bases. The region was amplified using primers OMBIRR for/OMBIRR rev, which complement sequences in the *dnaA* and *dnaN* genes located upstream and downstream of the right intergenic region. Consequently, this 247 bp *oriC* region included the 133 bp IRR, as well as 24 bp of the *dnaA* gene and 90 bp of the *dnaN* gene at either end. The pIRL45 plasmid (Fig. 1), which was unable to transform *M*. *bovis*, contained the intergenic region between the *recD* and *dnaA* genes and contained a 236 bp region with two AT rich regions of 30 bp and 16 bp and two DnaA boxes identical to the consensus TTATCCACA in *E*. *coli* and *B*. *subtilis*.

**Table 2:** Transformation efficiency of homologous and heterologous *oriC* plasmids^#^.

Plasmid	Origin*	Total size (kbp)	oriC region	*M*. *bovis* strain PG45			*M*. *agalactiae* strain PG2		
				Total (CFU/ml)^a^	TetR (CFU/ml)^b^	Efficiency^c^	Total (CFU/ml)^a^	TetR (CFU/ml)^b^	Efficiency^c^
**pWholeoriC45**	MBOVPG45	7.1	1.8 kbp	5.65×10^8^	4024	7.12 ×10^−6^	4.33×10^10^	5	1.16 ×10^−10^
**pIRR45**	MBOVPG45	5.5	247 bp	5.55×10^8^	17696	3.19 ×10^−5^	1.44×10^10^	1680	1.17 ×10^−7^
**pMM21-7**	MAG5632	9.5	1.3 kbp	8.30×10^8^	16	1.93 ×10^−8^	3.88×10^10^	2240	5.77 ×10^−8^
**p20-1miniO/T**	MAG5632	5.9	700 bp	6.90×10^8^	96	1.39 ×10^−7^	7.50×10^9^	9900	1.32 ×10^−6^
**pIRR5632**	MAG5632	5.5	219 bp	6.10×10^8^	1024	1.68×10^−6^	3.50×10^9^	7160	2.05 ×10^−6^
**pIRRPG2**	MAGPG2	5.5	217 bp	1.02×10^9^	214	2.09 ×10^−7^	3.14×10^10^	3760	1.20 ×10^−7^
**pIRL45**	MBOVPG45	5.5	239 bp	8.95×10^8^	0	0	NA	NA	NA

^#^From one representative experiment in which these plasmids were used to transform *M*. *bovis* and *M*. *agalactiae*

* MBOVPG45, *M*. *bovis* strain PG45; MAG5632, *M*. *agalactiae* strain 5632; MAGPG2, *M*. *agalactiae* strain PG2

NA: Not attempted

^a^Total concentration of viable organisms in colony forming units (CFU) without tetracycline selection

^b^Concentration of tetracycline resistant organisms after 2 h incubation at 37°C in non-selective broth

^c^Tetracycline resistant CFU/ total viable CFU (transformation efficiency)

Plasmids pIRR5632 and pIRRPG2, which were developed from *M*. *agalactiae* strains 5632 and PG2, respectively, were able to replicate in both *M*. *bovis* and *M*. *agalactiae*. The minimal *oriC* regions of *M*. *agalactiae* strains 5632 and PG2 were 219 bp and 217 bp, respectively, with each *oriC* region containing two AT rich regions of 17 bp each and a single DnaA box with a sequence similar to that of the consensus sequence (Fig. 1). As plasmid pIRR5632 replicated efficiently in *M*. *bovis* and *M*. *agalactiae* and had only a single DnaA box, an attempt was made to further reduce the size of the *oriC* region by generating a series of plasmids containing the DnaA box and the nearby AT rich regions (S1 Fig.), but all these plasmids failed to replicate in either species. This indicated that the minimal *oriC* region of *M*. *agalactiae* capable of initiating replication in these ruminant pathogens was 217 bp and included the 133 bp IRR together with 30 bp from the *dnaA* gene and 54 bp from the *dnaN* gene at either end.

### Efficiency of transformation with *oriC* plasmids in *M*. *bovis* and *M*. *agalactiae*

The pseudoresistant clones previously reported in studies using transposon mutagenesis in *M*. *bovis* [29] were not detected in the studies reported here. Therefore, it was possible to calculate the transformation efficiency, as there was no need to grow cultures in selective broth overnight before subculturing onto agar plates, which would have resulted in a biased calculation. There was a marked difference in transformation efficiency (Table 2) depending on the size and origin of the *oriC* plasmids. The transformation efficiency increased markedly with a decrease in the size of the *oriC* region in the construct, and, with the exception of pIRRPG2, homologous *oriC* plasmids were able to transform more efficiently than heterologous *oriC* plasmids. Plasmid pIRR5632 had a higher transformation efficiency in *M*. *bovis* (1.68×10^−6^) and in *M*. *agalactiae* (2.05×10^−6^). The larger pMM21-7 and pWholeoriC45 plasmids resulted in higher transformation efficiencies in homologous cultures, but with a decrease in size of the *oriC* region even heterologous plasmids were able to generate a considerable number of tetracycline resistant clones. Therefore, transformation efficiency was higher with homologous and smaller plasmids, although it was not possible to reduce the size of replicable *oriC* region beyond that of the smallest plasmids assessed here.

### Homologous recombination of the longer *oriC* plasmids in *M*. *bovis*

The presence of free, as well as integrated, plasmid in the transformants was demonstrated by Southern blot hybridisation using DIG-labelled *tetM*, *ampR* or *M*. *bovis* specific *oriC* probes. Two independent transformants generated using the pWholeoriC45 and the pMM21-7 plasmids in *M*. *bovis* strain PG45 were passaged by 1:5 dilution at each passage for up to 20 times in the presence of tetracycline and sampled at the 5^th^, 10^th^, 15^th^ and 20^th^ passage.

**Fig 2:**
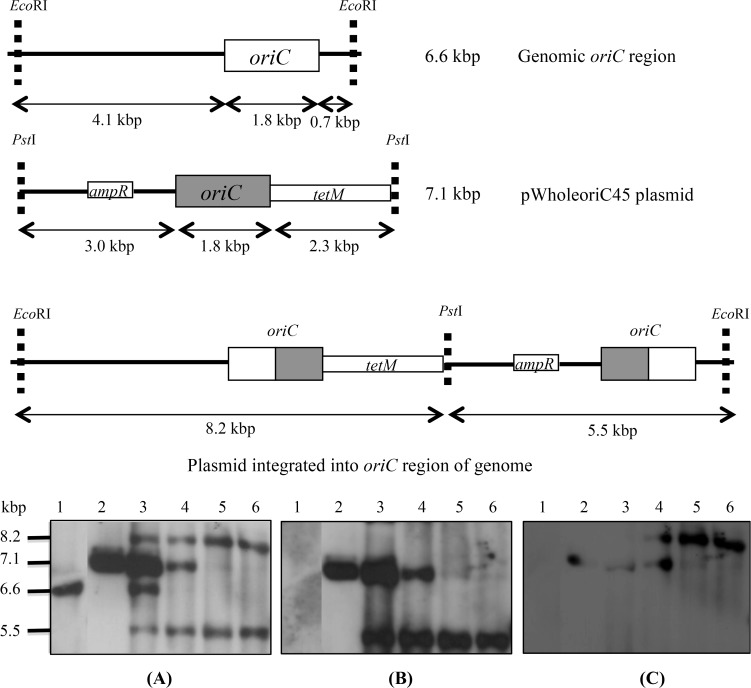
Chromosomal integration of pWholeoriC45 plasmid. Schematic diagram shows predicted integration of pWholeoriC45 plasmid into the genomic *oriC* region. Southern blot analysis of *EcoR*I-*Pst*I digested DNA probed with DIG-labelled probes targeting *oriC* region (Panel A), *ampR* (Panel B) and *tetM* (Panel C). Lane 1, untransformed *M*. *bovis*; lane 2, pWholeoriC45 plasmid; lanes 3 & 4, passage (p)5 and p10 showing the extrachromosomal plasmid as well as the integrated form; lanes 5 and 6, p15 and p20 showing complete integration of the plasmid into the chromosome.

The pWholeoriC45 plasmid had no *Eco*RI site and only a single *Pst*I site in the cloning site of pGEM-T, so *Eco*RI-*Pst*I digestion of the extrachromosomal form of the plasmid should yield a product of 7.1 kbp, while the chromosomal *oriC* in untransformed cells lies in an *Eco*RI fragment of 6.6 kbp. As pWholeoriC45 contained a unique *Pst*I site, plasmid integration via a single homologous recombination event at the chromosomal *oriC* locus was expected to result in two *oriC* copies that would be separated into two fragments upon digestion with *Eco*RI and *Pst*I, and their location could be further confirmed using *ampR* and *tetM* probes. In pWholeoriC45 transformants digested with a combination of *Eco*RI and *Pst*I (Fig. 2), the detection of 8.2 and 5.5 kbp fragments by the *oriC* probe indicated that integration of pWholeoriC45 into the chromosomal DNA had occurred. In contrast, the detection of 6.6 and 7.1 kbp fragments indicated that some of the transformed cells still contained the wild-type chromosomal *oriC* and free plasmid. At passage 5, the presence of four bands hybridising to the *oriC* probe suggested that a mixed population of cells was present. There was evidence of partial integration of the plasmid within the *oriC* region even at the 5^th^ passage in both of the transformants, and extrachromosomal plasmid was present till the 10^th^ passage in transformant 1 (Fig. 2, panel A) and the 15^th^ passage in transformant 2 (results not shown), after which no free plasmid could be detected. The blot was further probed with either the *ampR* or *tetM* probes. Hybridising fragments of 7.1 and 5.5 kbp were detected with the *ampR* probe (Fig. 2, panel B), and 7.1 and 8.2 kbp fragments with the *tetM* probe (Fig. 2, panel C), demonstrating the presence of both free and integrated forms of pWholeoriC45.

**Fig 3:**
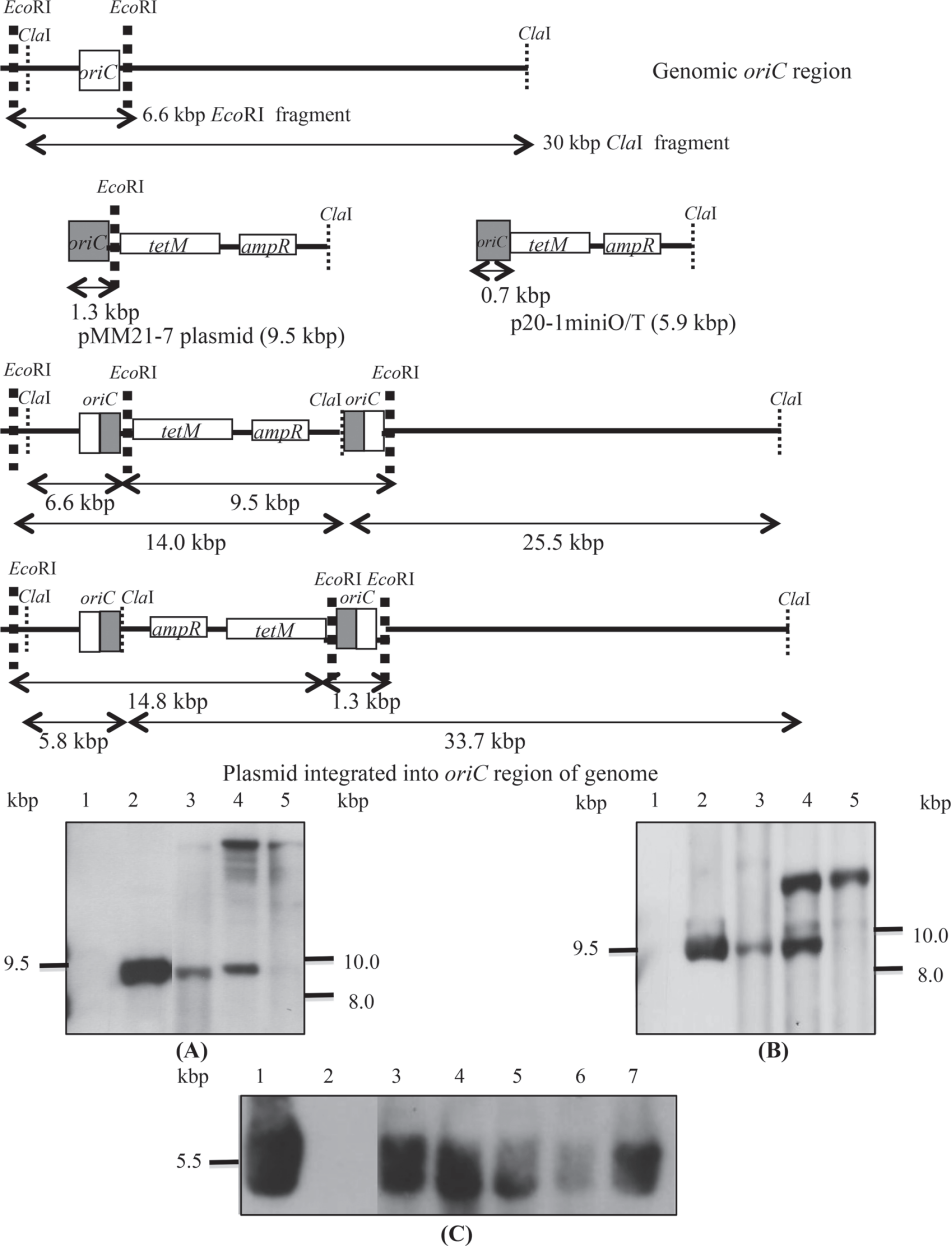
Chromosomal integration and stability of heterologous plasmids in *M*. *bovis*. Schematic representation of genomic *oriC* region, p20-1miniO/T and pMM21-7 plasmids and predicted organisation of the *oriC* region following integration of pMM21-7. Southern blot analysis of *Cla*I (panel A) or *Eco*RI (panel B) digested DNA probed with a DIG-labelled *ampR* probe. Lane 1, untransformed *M*. *bovis*; lane 2, pMM21-7; lane 3, clone 1 at passage 20; lane 4, clone 2 at p5 and lane 5, clone 2 at p10. Southern blot analysis of *Cla*I digested DNA probed with a DIG-labelled *ampR* probe (panel C). Lane 1, p20-1miniO/T; lane 2, untransformed *M*. *bovis* and lanes 3–7, p20-1miniO/T *M*. *bovis* transformants at p20.

The DNA of *M*. *bovis* pMM21-7 transformants was digested with either *Cla*I or *Eco*RI and hybridised with the *ampR* probe. In clone 1, plasmid was maintained extrachromosomally till the 20^th^ passage, while in clone 2 both free and integrated forms of plasmids were visible at the 5^th^ passage, and by the 10^th^ passage the plasmid appeared to have completely integrated into the genome (Fig. 3, panels A and B).

### Stability of smaller *oriC* plasmids in *M*. *bovis*

**Fig 4:**
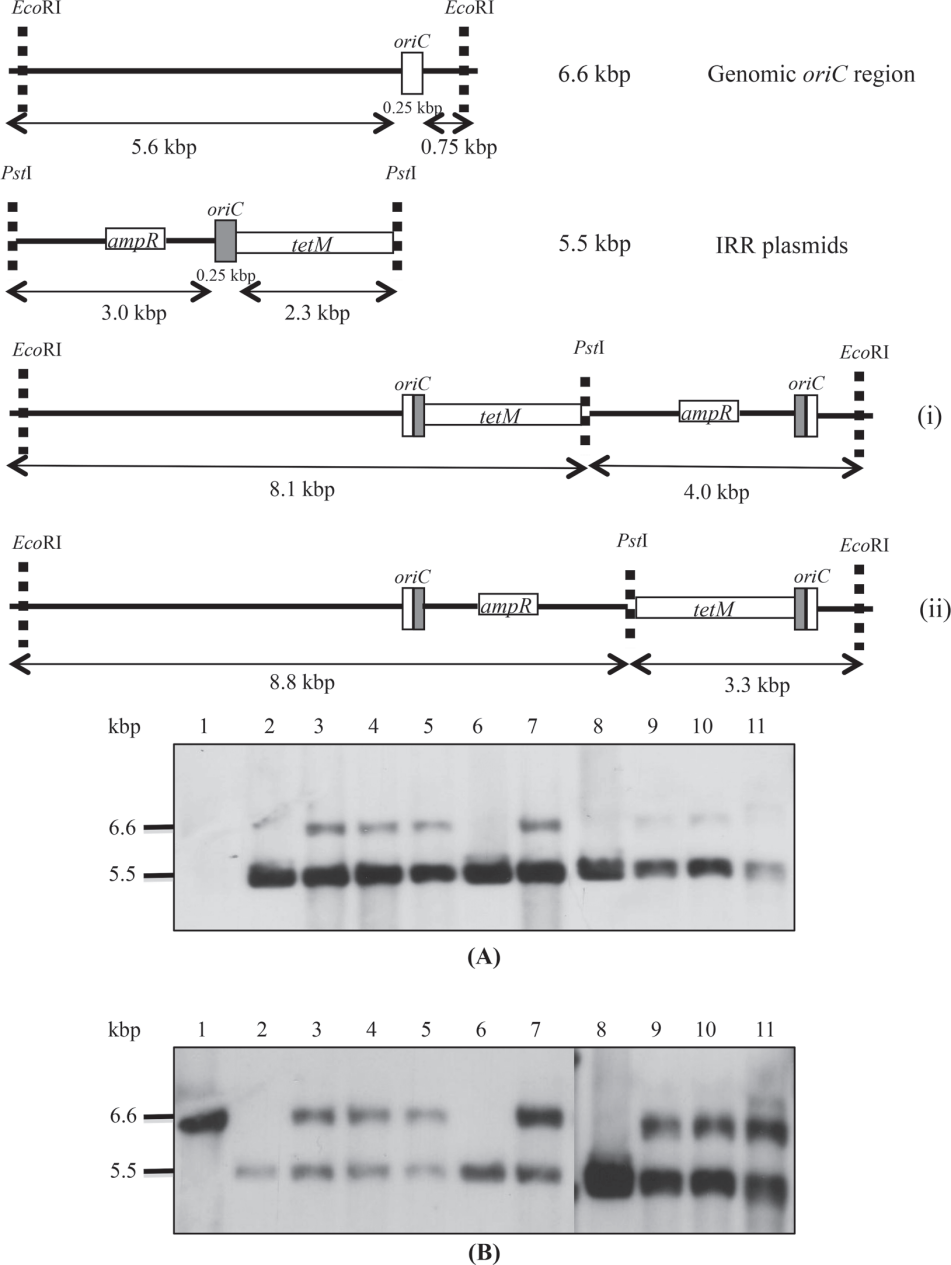
Stability of smaller *oriC* plasmids in *M*. *bovis*. Schematic representation of genomic *oriC* region, IRR plasmids and predicted organisation of the *oriC* region following integration of different IRR plasmids. Southern blot analysis of *EcoR*I-*Pst*I digested DNA probed with DIG-labelled probes targeting *ampR* (panel A) and *oriC* region (panel B). Smaller *oriC* plasmids were stable in *M*. *bovis* till p15-p20, but some integration appeared to have occurred in the *oriC* region. Lane 1, untransformed *M*. *bovis*; lanes, 2, 6 and 8, pIRR5632, pIRRPG2 and pIRR45, respectively. Lanes 3–5: pIRR5632 *M*. *bovis* transformants at p10, p15 and p20; lane 7, pIRRPG2 *M*. *bovis* transformants at p15; lanes 9–11, pIRR45 *M*. *bovis* transformants at p10, p15 and p20.

To evaluate the stability of the smaller homologous and heterologous plasmids in *M*. *bovis*, three or more independent transformants obtained with each of the plasmids p20-1miniO/T, pIRR45, pIRRPG2 and pIRR5632 were passaged. The DNA of p20-1miniO/T transformants was digested with *Cla*I, while that of transformants with plasmids based on the IRR region were digested with *Eco*RI and *Pst*I. In contrast to the transformants obtained with the larger *oriC* plasmids, transformants containing the p20-1miniO/T (Fig. 3, panel C) and smaller IRR based *oriC* plasmids had only a single hybridising band (Fig. 4, panel A), corresponding to the size expected for the free plasmid, when probed with either *tetM* or *ampR* gene fragments, whilst two bands, corresponding to free IRR based plasmid and the intact chromosomal *oriC* region, were detected when they were hybridised with the *oriC* probe (Fig. 4, panel B). All the randomly selected clones stably maintained the *oriC* plasmids till the 20^th^ passage. The *oriC* probe generated strong hybridisation signals with the chromosomal *oriC* region as well as the extrachromosomal plasmid, while the *tetM* and *ampR* probes bound very weakly to the chromosomal *oriC* region in some clones, in addition to the intense signal from bands corresponding to extrachromosomal plasmid. This may suggest a low level of integration of the plasmids at the *oriC* region, but could not be explained by a single crossover event, as this would yield predicted hybridising bands of 4.0 or 8.8 kbp with the *ampR* probe.

### Assessment of gene disruption by homologous recombination

In an attempt to inactivate gene targets, engineered constructs containing internal fragments of the *xer1*, *p48* or type II restriction endonuclease genes were generated (S2 Fig.). The *recA* gene of *M*. *gallisepticum* strain S6, along with its promoter, was incorporated into the plasmid in order to promote homologous recombination. The *oriC* plasmids based on the IRR region were chosen for gene disruption as these were found to be present extrachromosomally till the 20^th^ passage. Following electroporation of these plasmids into *M*. *bovis*, 3 to 5 clones were selected from each transformation and passaged 10 times at a 1:9 dilution each passage. The *M*. *gallisepticum recA* gene contained an *Eco*RI site that was unique within the plasmids, so DNA of transformants obtained using the engineered constructs were digested with *Eco*RI. Southern blot analysis was performed using the *ampR* probe. The *oriC* plasmids containing the internal gene fragments and the heterologous *recA* replicated as free plasmids in the transformants. The *ampR* probe bound to a 7.4 kbp band, indicating the presence of extrachromosomal plasmid, and a 9.4 kbp band (results not shown), corresponding to the predicted size of the fragment following integration of the plasmids into the chromosomal *oriC* region of *M*. *bovis*. However, no bands indicative of homologous recombination with the target genes were seen.

## Discussion

In most bacteria, *oriC* is located in the vicinity of *dnaA* (S3 Fig.), and DNA replication starts with the specific interaction of DnaA with the 9 bp DnaA boxes in the *oriC* region. Consensus sequences (TTATCCACA; DnaA boxes) have been identified in the intergenic regions flanking the *dnaA* gene in mollicutes [31]. Replicable plasmids based on *oriC* regions have been successfully developed, including pMM21-7, which contains a 1.3 kbp *oriC* region, and its shorter derivative p20-1miniO/T, in *M*. *agalactiae* [20,28]. In the present study, *M*. *bovis* was initially transformed with the pWholeoriC45 plasmid, containing *dnaA* and upstream and downstream intergenic regions, making it the first homologous *oriC* plasmid capable of replicating in *M*. *bovis*. Moreover, *M*. *bovis* PG45 could be successfully transformed with the heterologous pMM21-7 *oriC* plasmid originally developed for *M*. *agalactiae* and its derivative p20-1miniO/T. Similarly, the pWholeoriC45 plasmid developed from *M*. *bovis* could be used to transform *M*. *agalactiae* cells. This clearly indicated that heterologous plasmids developed from either *M*. *bovis* or *M*. *agalactiae* were able to replicate in either species.

Host specificity studies in mycoplasmas have suggested that the host range of *oriC* plasmids is restricted to closely related species [31]. The *oriC* plasmids of *M*. *gallisepticum* and *M*. *imitans* have been shown to be able to replicate in both species [15]. Similarly, *oriC* plasmids derived from *M*. *mycoides* subspecies *mycoides* and *M*. *mycoides* subspecies *capri* are able to replicate in each other, as well as in *M*. *capricolum* [19]. Conversely, attempts to transform *M*. *agalactiae* and members of the mycoides cluster with *oriC* plasmids derived from *M*. *pulmonis* were unsuccessful [19,28]. The nucleotide sequence of the replicable *oriC* region and the DnaA protein sequences of *M*. *pulmonis* are distinct from those of *M*. *agalactiae* and members of the mycoides cluster. A phylogenetic tree based on DnaA protein sequences of mycoplasmas (S4 Fig.) shows that there are close relationships between the DnaA proteins of *M*. *bovis* and *M*. *agalactiae*, between those of *M*. *gallisepticum* and *M*. *imitans*, and between those of various species in the mycoides cluster, including *M*. *mycoides* subspecies *mycoides*, *M*. *mycoides* subspecies *capri* and *M*. *capricolum*. The nucleotide sequences of the *oriC* regions in each of these groups of species exhibit high levels of similarity, which is probably one of the reasons for the compatibility of *oriC* plasmids derived from one species with other closely related species. The present study supports this hypothesis. The replicable *oriC* regions of *M*. *bovis* and *M*. *agalactiae* have more than 80% nucleotide sequence identity (S5 Fig.) and more than 95% DnaA protein identity. This could explain why *M*. *bovis* and *M*. *agalactiae* can support replication of their heterologous *oriC* plasmids.

To identify the smallest *oriC* region capable of replicating in *M*. *bovis* and *M*. *agalactiae*, a series of *oriC* plasmid constructs were developed. Only the plasmids containing the region downstream of *dnaA* could be detected in *M*. *bovis* and *M*. *agalactiae*, indicating that the AT rich sequences found in this region were essential for plasmid replication, even though the region upstream from the *dnaA* gene of *M*. *bovis* included two DnaA boxes with similar consensus sequences. This suggests that, at least in *M*. *bovis* and *M*. *agalactiae*, *dnaA* and the region upstream of it are not essential or sufficient for replication of *oriC* plasmids.

The minimal functional *oriC* region of *M*. *bovis* determined in this study was 247 bp in size, while those in *M*. *agalactiae* strains 5632 and PG2 were 219 bp and 217 bp, respectively; these regions are all located downstream of the *dnaA* gene. In two other members of the hominis phylogenetic group, *M*. *pulmonis* [22] and *M*. *hyopneumoniae* [32], the minimal region necessary for replication of an *oriC* plasmid devoid of *dnaA* contained both the upstream and downstream *dnaA* flanking intergenic regions.

The regions downstream of *dnaA* in *M*. *bovis* and *M*. *agalactiae* contain two DnaA boxes and a single DnaA box, respectively. It has been reported previously that only a few DnaA boxes and an AT rich region were sufficient for replication in mycoplasmas. For instance, a functional *oriC* vector for *M*. *gallisepticum* strain S6 contains two DnaA boxes and two AT rich clusters [15], and that for *S*. *citri* contains three DnaA boxes and two AT rich regions [33].

As the intergenic region downstream of *dnaA* in *M*. *agalactiae* strain 5632 contains a single DnaA box, the size of the *oriC* construct was reduced further. A number of smaller versions of *oriC* plasmids based on the replicable pIRR5632 plasmid were produced, but all failed to replicate in *M*. *bovis* or *M*. *agalactiae*. This showed that the intergenic region downstream of *dnaA* alone is not sufficient for optimal replication of *oriC* plasmids. In *Mycobacterium smegmatis*, it was found that replication of *oriC* plasmids lacking regions from either the 5’ end or 3’ end of adjacent genes was greatly reduced. It has been concluded that the 3’ end of *dnaA* participates in chromosomal partitioning and is therefore essential for optimal activity of *oriC* plasmids in *M*. *smegmatis*, as well as in *B*. *subtilis* [34,35]. In *S*. *citri*, an *oriC* based plasmid containing three DnaA boxes but only one AT rich region failed to replicate, indicating that the AT cluster downstream of the DnaA boxes is essential for plasmid replication [33]. All *oriC* plasmids in the present study were generated with a pGEM-T backbone, and further examination of pGEM-T revealed at least 4 DnaA boxes, with the nucleotide sequence of one box exactly the same as the consensus sequence, further confirming that DnaA boxes alone are not sufficient for initiation of replication and that the location and size of the AT rich clusters, relative to DnaA boxes, is critical.

The transformation efficiency was found to be higher for homologous and smaller *oriC* plasmids. These results were in agreement with earlier studies that showed that a decrease in the size of the *oriC* region in a homologous plasmid resulted in higher transformation efficiencies in *M*. *agalactiae* [28]. The higher transformation efficiency observed with homologous plasmids compared to heterologous plasmids could be related to differences between the AT rich regions. It has been demonstrated in *M*. *smegmatis* that an AT rich sequence of a particular length is important for the function of an *oriC*, and that variation in the length of this AT rich region influenced transformation efficiency, and that even an increase in the length of the AT rich sequence was inhibitory [35].

In this study, the longer *oriC* regions of both homologous and heterologous plasmids readily integrated into the *M*. *bovis* chromosome. This has also been described in *M*. *gallisepticum* [15], *S*. *citri* [36] and *M*. *pulmonis* [22]. In order to produce *oriC* plasmids that would not integrate readily into the chromosome and could be used for targeted gene disruption, smaller constructs based on the IRR regions of *M*. *bovis* PG2 and *M*. *agalactiae* 5632 were produced and, following transformation, were found to be stable and remain in an extrachromosomal location up to the 20^th^ passage. Targeted gene disruption was attempted in *M*. *bovis* using IRR based *oriC* plasmids. Recombination events in mollicutes are dependent on the *recA* gene [13,37]. The *recA* gene from *E*. *coli* has been used in *M*. *mycoides* subspecies *capri* to generate knockout mutants [38]. The heterologous *recA* gene of *M*. *gallisepticum* strain S6 incorporated into our constructs was expected to promote homologous recombination, while having sufficient nucleotide sequence difference (with only 67% sequence identity between nucleotide 933 and 1342 bp) to reduce the likelihood of recombination with the *M*. *bovis recA*. However, our attempts to disrupt gene targets using stable *oriC* plasmids in *M*. *bovis* failed, with most plasmids integrating into the *oriC* region of the chromosome by the 10^th^ passage. While we were unable to confirm expression of the heterologous RecA as we lacked reagents to specifically detect its expression, the inclusion of the heterologous *recA* probably promoted homologous recombination within the *oriC* region, as plasmids devoid of *recA* were maintained in an extrachromosomal form up to the 20^th^ passage. Although replicable *oriC* plasmids have been successfully used to inactivate several gene targets in mollicutes, recombination at the target gene site is never straightforward. In most cases, the disruption vector integrates into the host chromosome in the *oriC* region, rather than at the target gene site, as was the case in our study. In some cases, *oriC* plasmids have been shown to disrupt the targeted gene, but the mutant containing the disrupted gene could not be isolated [22], and, in a few other cases, the plasmids have integrated randomly through illegitimate recombination, rather than through homologous recombination in the *oriC* region or in the gene targets [15]. Internal fragments of gene targets varying in size from 251 to 462 bp nucleotides were included in the plasmids. The size of these fragments was comparable to that of the *oriC* region, so there appeared to be a preference for the plasmid *oriC* region to recombine with the *oriC* region of the *M*. *bovis* chromosome rather than for the internal gene fragment to recombine with the target gene. It may be that binding of DnaA to *ori*C results in more stable single stranded regions for initiation of recombination than can be generated at the targeted genes by the heterologous RecA we included in the plasmids. In addition, the GC content of the *oriC* regions in the plasmids were 19 and 20%, while that of the internal fragment of *p48* was 35%, and those of type II restriction endonuclease gene and *xer1* were 27%. The lower GC content of the *oriC* region may thus have also contributed to the preferential recombination at the oriC region. It is possible that use of a larger internal fragment of the target genes may have enhanced recombination in the intended target.

Although specific gene targets were unable to be disrupted, the stable extrachromosomal plasmids developed in this study could also be used in *M*. *bovis* for complementation of disrupted genes function in transposon mutants and for expression of foreign genes.

## Summary

This study has developed and demonstrated the potential of *oriC* vectors as genetic tools in two important ruminant mycoplasmas, *M*. *bovis* and *M*. *agalactiae*. The resistance marker, *tetM*, was found to be functional in both free and integrated forms of the plasmids.

## Supporting Information

S1 Fig Development of *M*. *agalactiae* plasmids with minimal *oriC* regions. The single DnaA box located between nt 120 and 128 is shown above the sequence. Four different regions, of 133 bp (intergenic region), 90 bp, 66 bp or 38 bp, all including the DnaA box and putative AT rich regions, were amplified from pIRR5632. The amplified products were ligated separately into the multicloning site of pGEM-T (Promega) and the tetracycline resistance gene (*tetM*) was cloned into the *Spe*I restriction endonuclease cleavage site of these plasmids.
(TIF)

S2 Fig Development of engineered constructs for disrupting gene targets. Internal fragments of the *p48* (lane 1, 392 bp), *type II restriction endonuclease* (lane 2, 462 bp) and *xer1* (lane 3, 251 bp) genes were amplified from *M*. *bovis* strain PG45 with appropriate primers and inserted between the *Not*I and *Pst*I sites of the IRR based *oriC* plasmid. To promote homologous recombination, the *recA* gene was amplified from *M*. *gallisepticum* strain S6 and cloned between the *Pst*I and *Sal*I cleavage sites of the construct.
(TIF)

S3 Fig Gene order surrounding the chromosomal *oriC* regions of *Mycoplasma* species and the structure of the putative *oriC* regions. Triangles indicate the location of the DnaA boxes and shaded rectangles indicate the location of AT rich regions. Adapted and modified from Lee *et al*. (2008).
(TIF)

S4 Fig Neighbour joining inference of phylogenetic relationships based on DnaA protein sequences. DnaA protein sequences of various mycoplasmas were obtained from Molligen and NCBI databases and used to produce ClustalW alignments. The phylogenetic tree was constructed using the Jukes-Cantor parameters for neighbour joining inference implemented in the Geneious tree builder, with *Bacillus subtilis* as an outgroup. The bootstrap values indicated on the tree were obtained from 5000 replicates.
(TIF)

S5 Fig Alignment of the replicable *oriC* regions of *M*. *bovis* PG45 (MBOVPG45), and *M*. *agalactiae* strains PG2 (MAGPG2) and 5632 (MAG5632), contained in the pIRR45, pIRRPG2 and pIRR5632 plasmids. The *M*. *bovis* PG45 region had 75.4 and 73.1% similarity to *M*. *agalactiae* strains PG2 and 5632, respectively, while the similarity between the two *M*. *agalactiae* strains was 86.6%. The boxed regions indicate the location of the DnaA boxes, 2 in *M*. *bovis* PG45 and one in *M*. *agalactiae*.
(TIF)
