# Correction: Development and Host Compatibility of Plasmids for Two Important Ruminant Pathogens, *Mycoplasma bovis* and *Mycoplasma agalactiae*

**DOI:** 10.1371/journal.pone.0125268

**Published:** 2015-04-10

**Authors:** Shukriti Sharma, Chistine Citti, Eveline Sagné, Marc S. Marenda, Philip F. Markham, Glenn F. Browning

S2 Fig, “Development of engineered constructs for disrupting gene targets” is an incorrect duplicate of Fig 2. Please view the correct S2 Fig below.

## Supporting Information

S2 Fig Development of engineered constructs for disrupting gene targets. Internal fragments of the p48 (lane 1, 392 bp), type II restriction endonuclease (lane 2, 462 bp) and xer1 (lane 3, 251 bp) genes were amplified from M. bovis strain PG45 with appropriate primers and inserted between the NotI and PstI sites of the IRR based oriC plasmid. To promote homologous recombination, the recA gene was amplified from M. gallisepticum strain S6 and cloned between the PstI and SalI cleavage sites of the construct.
(PPT)
