# Supplementary figures and images for: Development and Host Compatibility of Plasmids for Two Important Ruminant Pathogens, Mycoplasma bovis and Mycoplasma agalactiae
Source: PLoS One. 2015 Mar 6;10(3):e0119000. doi: 10.1371/journal.pone.0119000 (PMC4351888; doi:10.1371/journal.pone.0119000)

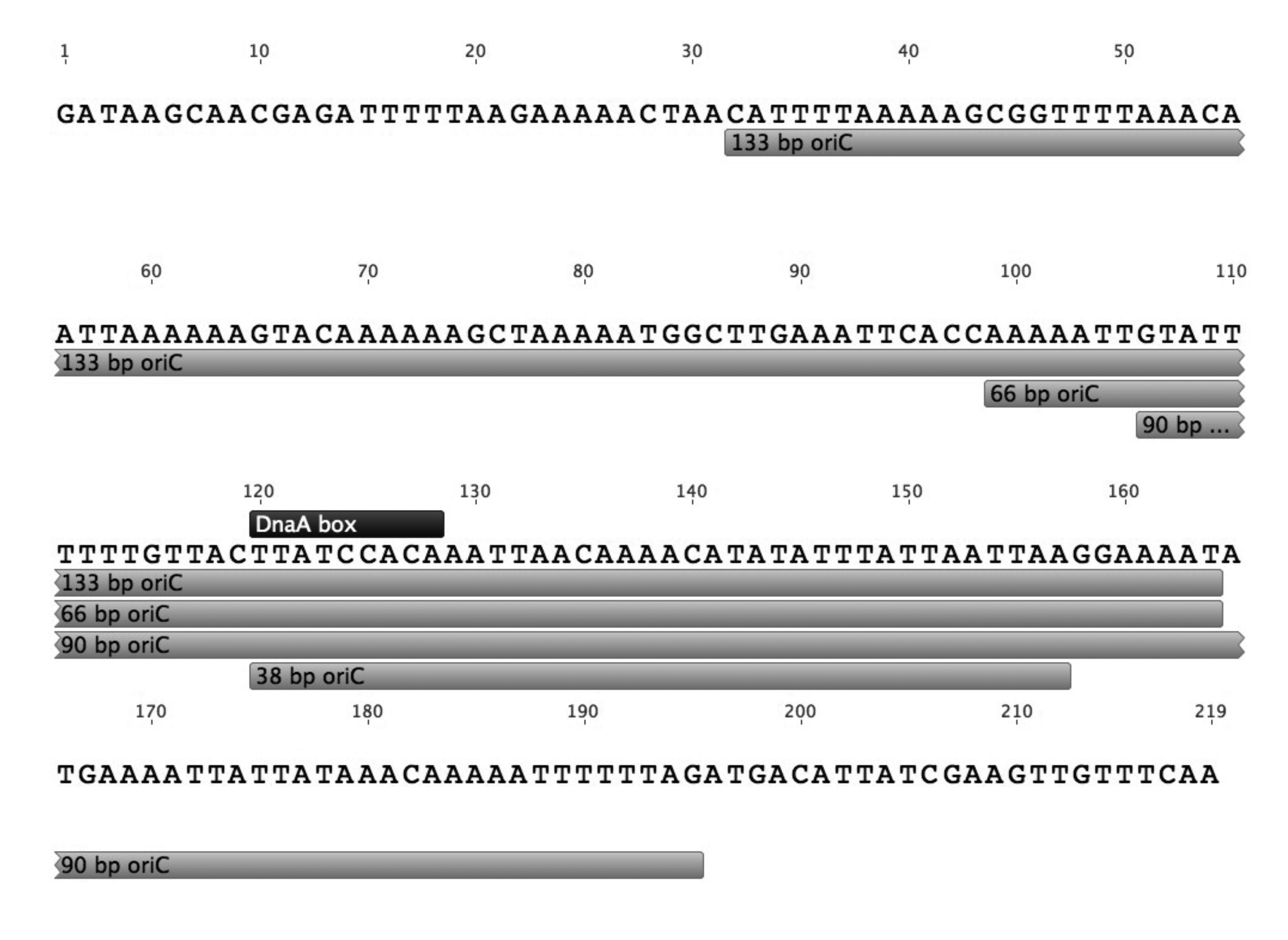

Supplement: S1 Fig — The single DnaA box located between nt 120 and 128 is shown above the sequence. Four different regions, of 133 bp (intergenic region), 90 bp, 66 bp or 38 bp, all including the DnaA box and putative AT rich regions, were amplified from pIRR5632. The amplified products were ligated separately into the multicloning site of pGEM-T (Promega) and the tetracycline resistance gene (tetM) was cloned into the SpeI restriction endonuclease cleavage site of these plasmids. (TIF) [file pone.0119000.s001.tif]

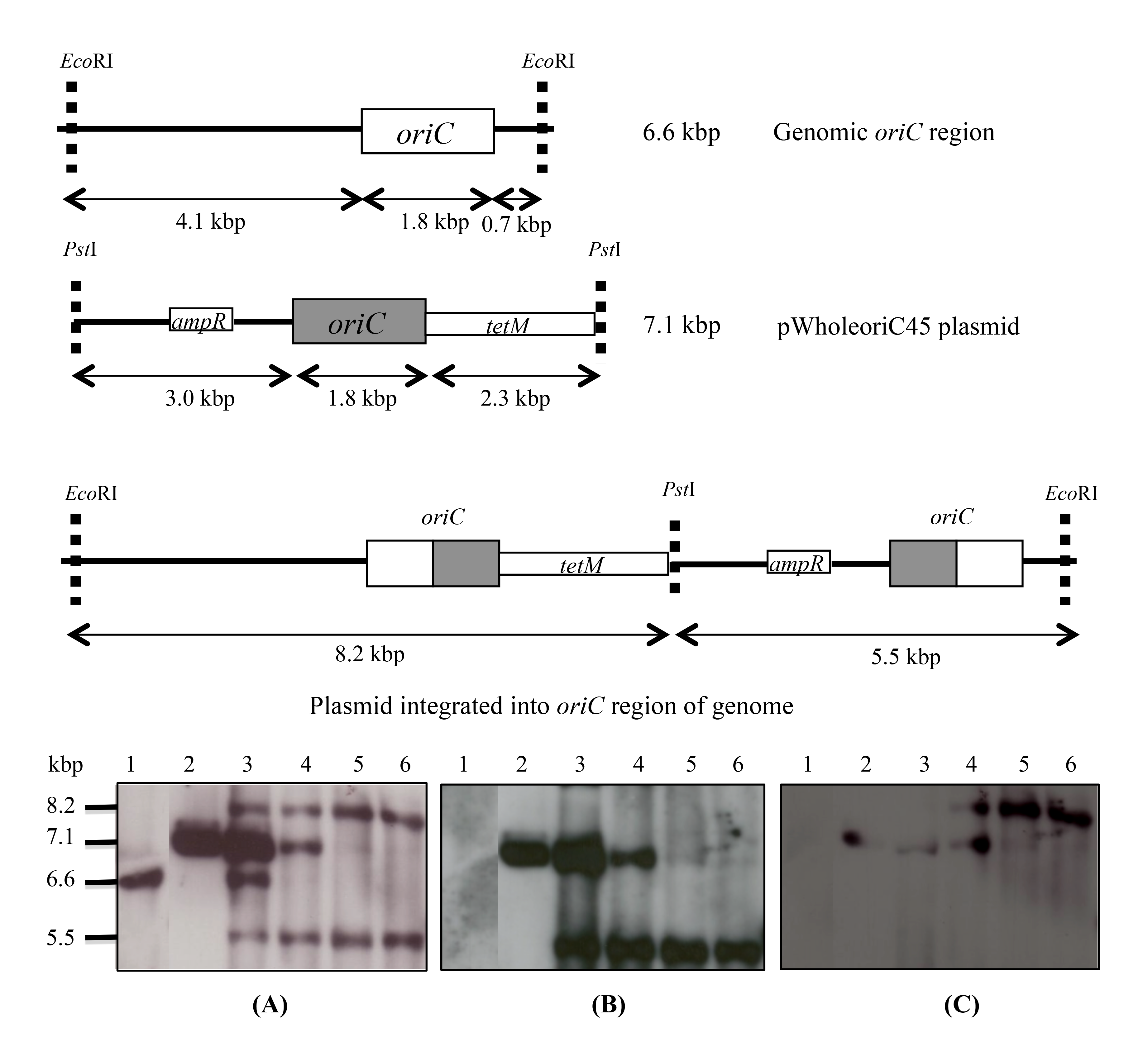

Supplement: S2 Fig — Internal fragments of the p48 (lane 1, 392 bp), type II restriction endonuclease (lane 2, 462 bp) and xer1 (lane 3, 251 bp) genes were amplified from M. bovis strain PG45 with appropriate primers and inserted between the NotI and PstI sites of the IRR based oriC plasmid. To promote homologous recombination, the recA gene was amplified from M. gallisepticum strain S6 and cloned between the PstI and SalI cleavage sites of the construct. (TIF) [file pone.0119000.s002.tif]

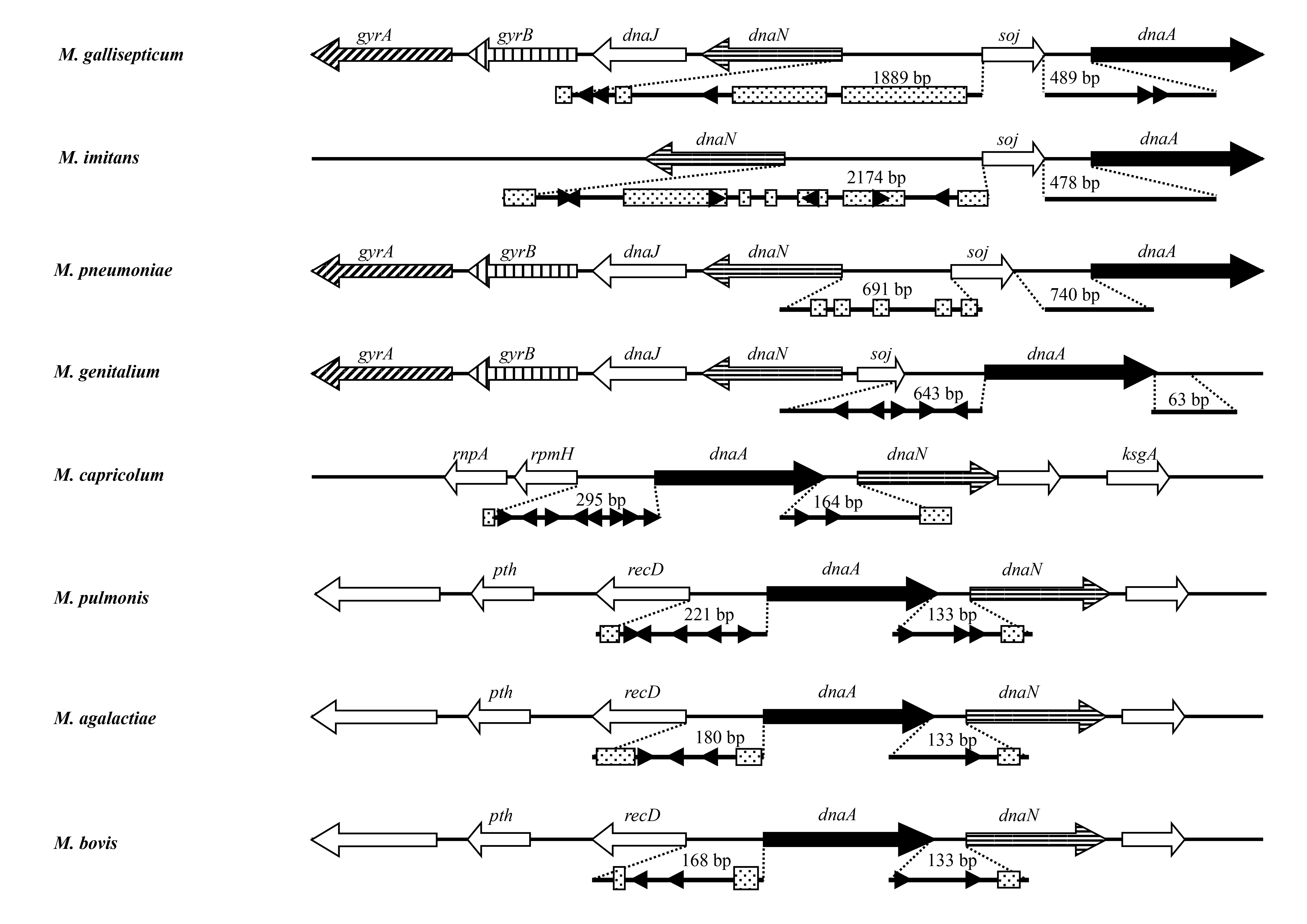

Supplement: S3 Fig — Triangles indicate the location of the DnaA boxes and shaded rectangles indicate the location of AT rich regions. Adapted and modified from Lee et al. (2008). (TIF) [file pone.0119000.s003.tif]

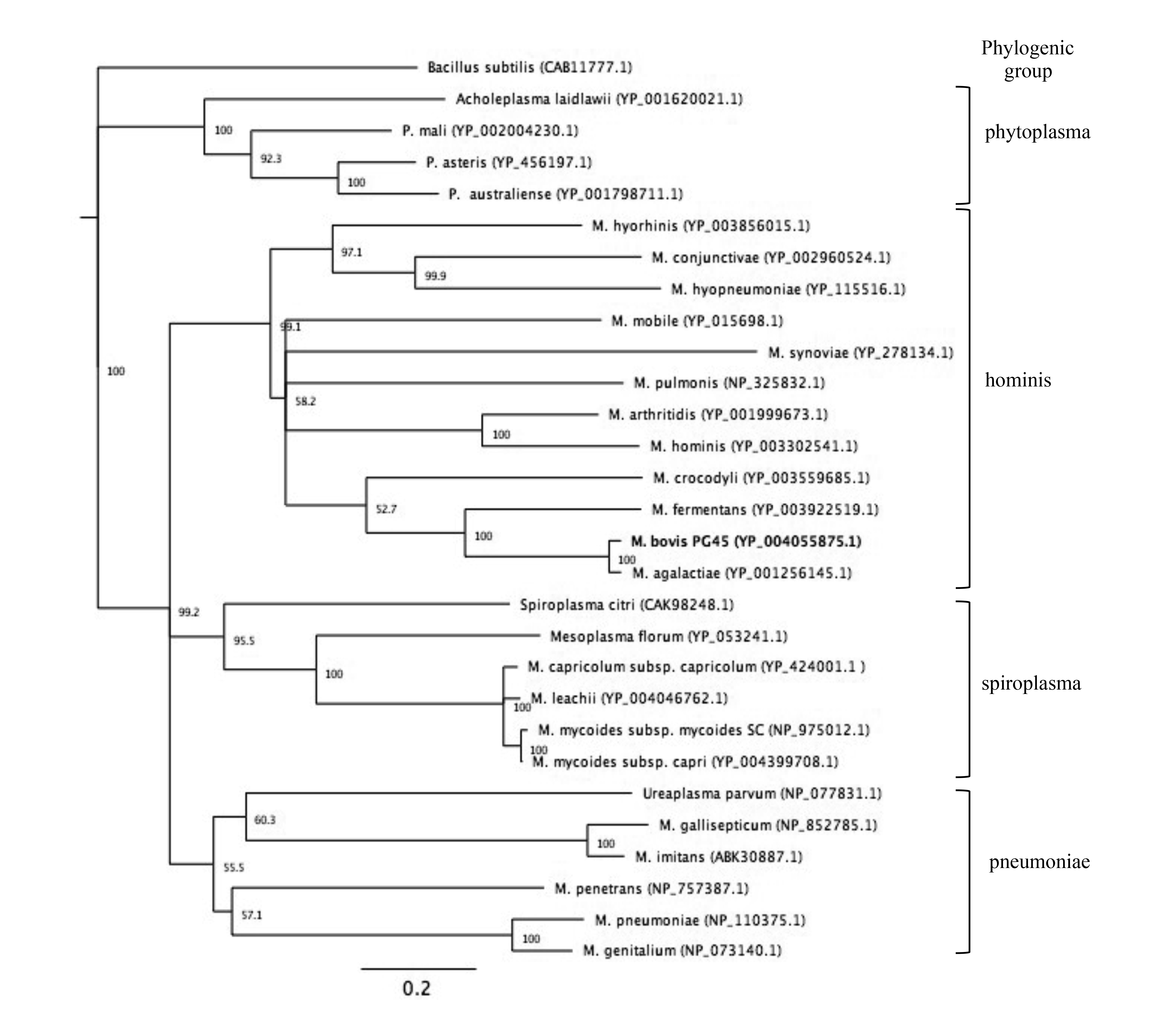

Supplement: S4 Fig — DnaA protein sequences of various mycoplasmas were obtained from Molligen and NCBI databases and used to produce ClustalW alignments. The phylogenetic tree was constructed using the Jukes-Cantor parameters for neighbour joining inference implemented in the Geneious tree builder, with Bacillus subtilis as an outgroup. The bootstrap values indicated on the tree were obtained from 5000 replicates. (TIF) [file pone.0119000.s004.tif]

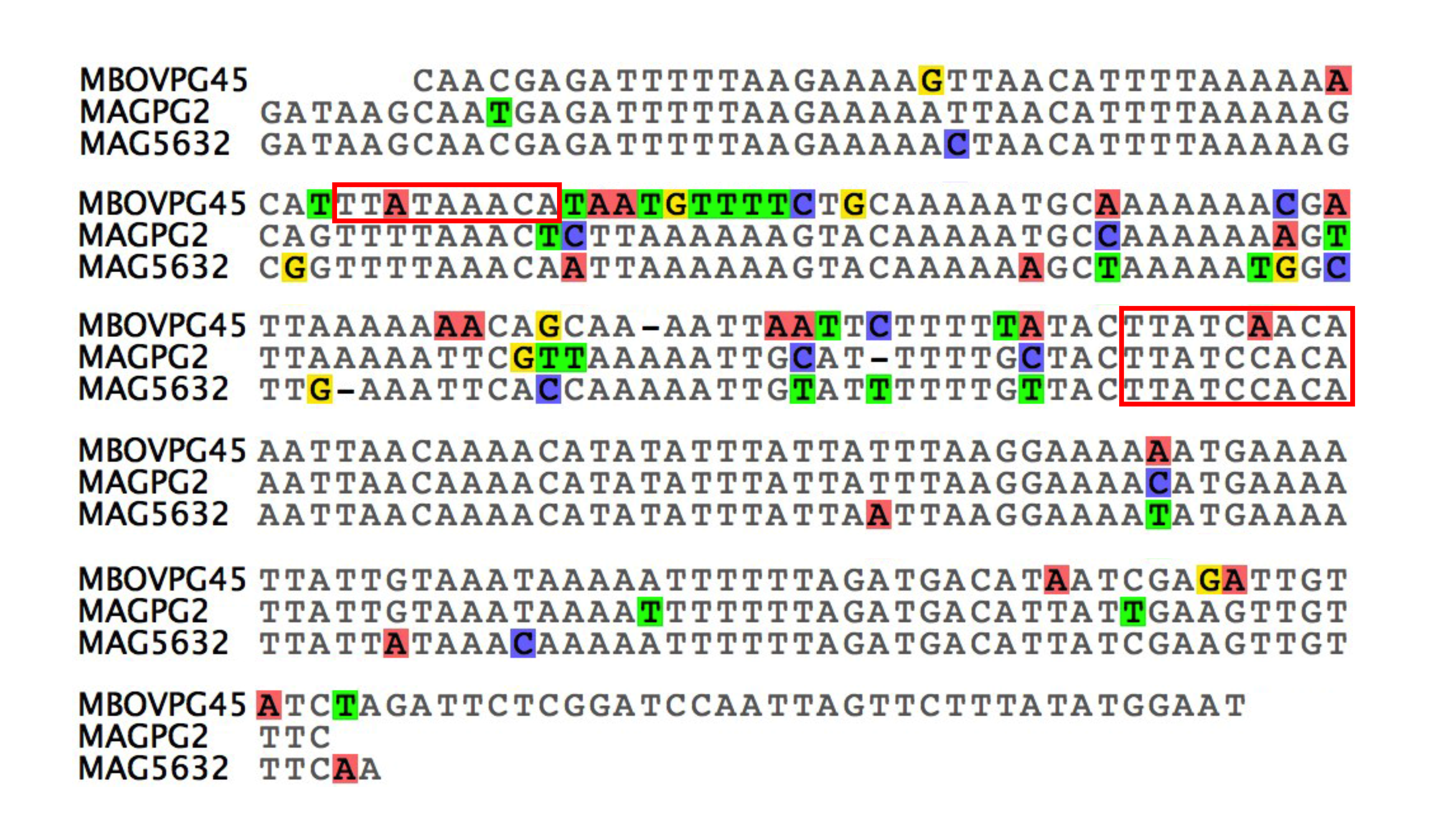

Supplement: S5 Fig — The M. bovis PG45 region had 75.4 and 73.1% similarity to M. agalactiae strains PG2 and 5632, respectively, while the similarity between the two M. agalactiae strains was 86.6%. The boxed regions indicate the location of the DnaA boxes, 2 in M. bovis PG45 and one in M. agalactiae. (TIF) [file pone.0119000.s005.tif]
