# Supplementary material for: Correction: Development and Host Compatibility of Plasmids for Two Important Ruminant Pathogens, Mycoplasma bovis and Mycoplasma agalactiae
Source: PLoS One. 2015 Apr 10;10(4):e0125268. doi: 10.1371/journal.pone.0125268 (PMC4393319; doi:10.1371/journal.pone.0125268)
Supplement: S2 Fig — Internal fragments of the p48 (lane 1, 392 bp), type II restriction endonuclease (lane 2, 462 bp) and xer1 (lane 3, 251 bp) genes were amplified from M. bovis strain PG45 with appropriate primers and inserted between the NotI and PstI sites of the IRR based oriC plasmid. To promote homologous recombination, the recA gene was amplified from M. gallisepticum strain S6 and cloned between the PstI and SalI cleavage sites of the construct. (PPT) [file pone.0125268.s001.ppt]

## Slide 1
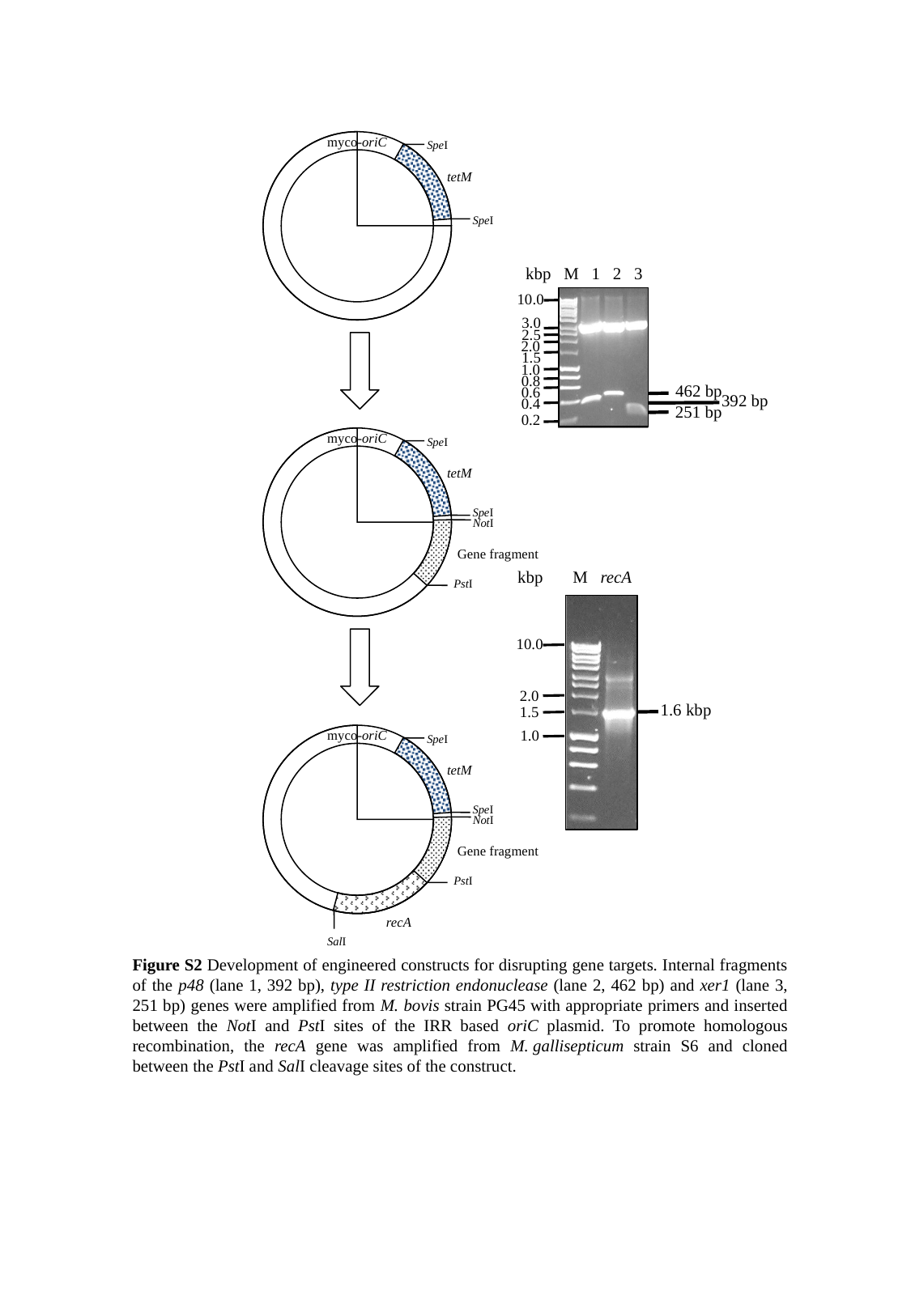

SpeI
myco-oriC
SpeI
tetM
kbp M 1 2 3
10.0
3.0
2.5
2.0
1.5
1.0
0.8
462 bp
 392 bp
251 bp
0.6
0.4
0.2
SpeI
myco-oriC
tetM
SpeI
NotI
Gene fragment
PstI
kbp M recA
10.0
2.0
1.5
1.0
1.6 kbp
SpeI
myco-oriC
tetM
SpeI
NotI
Gene fragment
PstI
recA
SalI
Figure S2 Development of engineered constructs for disrupting gene targets. Internal fragments of the p48 (lane 1, 392 bp), type II restriction endonuclease (lane 2, 462 bp) and xer1 (lane 3, 251 bp) genes were amplified from M. bovis strain PG45 with appropriate primers and inserted between the NotI and PstI sites of the IRR based oriC plasmid. To promote homologous recombination, the recA gene was amplified from M. gallisepticum strain S6 and cloned between the PstI and SalI cleavage sites of the construct.
